# The Role of Noninvasive Techniques in Stroke Therapy

**DOI:** 10.1155/2008/672582

**Published:** 2007-12-11

**Authors:** Daniel Maxwell Bernad, Julien Doyon

**Affiliations:** ^1^Institute of Biotechnology University of Cambridge, Tennis Court Road, Cambridge CB2 3HU, UK; ^2^Department of Medicine, University of Ottawa, 451 Smyth Road, Ottawa, Ontario K1H 8M5, Canada; ^3^Functional Neuroimaging Unit, University of Montreal Geriatric Institute, 4565, Queen-Mary Street, Montreal, Quebec H3W 1W5, Canada

## Abstract

Noninvasive techniques such as functional magnetic resonance imaging (fMRI) and transcranial magnetic stimulation (TMS) have provided insight into understanding how neural connections are altered in consequence to cerebrovascular injury. The first part of this review will briefly survey some of the methodological issues and limitations related to noninvasive poststroke motor recovery studies. The second section will investigate some of the different neural mechanisms that underlie neurorehabilitation in stroke patients. The third part will explore our current understanding of motor memory processing, describe the neural structures that subserve motor memory consolidation, and discuss the current literature related to memory reconsolidation in healthy adults. Lastly, this paper will suggest the potential therapeutic applications of integrating noninvasive tools with memory consolidation and reconsolidation theories to enhance motor recovery. The overall objective of this work is to demonstrate how noninvasive technologies have been utilized in the multidisciplinary field of clinical behavioral neuroscience and to highlight their potential to be employed as clinical tools to promote individualized motor recovery in stroke patients.

## 1. INTRODUCTION AND OUTLINE

Stroke is a debilitating disorder that has the potential to cause substantial sensory, motor, and cognitive impairment. Roughly half of all stroke victims will have some degree of residual motor impairment [1], becoming partially dependent for performing activities of daily living [2]. Acute stroke onset, usually defined as the first six to twelve hours following the interruption of blood supply to neurons, results in a core of dead neurons surrounded by a penumbra where neural tissue remains dysfunctional. If blood flow is not reinstated, the neurons within the penumbra will die and clinical deficits will tend to stabilize. Research efforts have focused on understanding the mechanisms that subserve plasticity or functional modifications in consequence to neuronal damage. Modifications in neural connections and networks are believed to result from cellular or synaptic changes in neuronal functioning following injury. To this extent, a framework describing neurorehabilitation can be conceptualized from a cellular, systems, and behavioral perspective [3]. For example, changes that occur at a cellular level can initiate different molecular mechanisms such as engaging remyelination. Adaptation that arises at a systems level can involve the recruitment of new neural regions, which can activate the same final output pathway. In addition, behavioral level changes can result from enhanced motivation or altered cognitive strategies to regain function by completing a particular motor task [3]. Diagnostic tools that can detect early stroke onset is crucial for neuronal survival and provides clinicians and rehabilitation specialists with a wider range of treatment options, which ultimately is more effective in helping patients recover motor function.

Recent advances in noninvasive imaging techniques have enabled physicians to diagnose stroke at an earlier point in time and have provided greater comprehension of the changes in neural activity that transpire after stroke [3]. In particular, the application of functional magnetic imaging (fMRI) and transcranial magnetic stimulation (TMS) has provided a better understanding of the neural substrates that subserve recovery. The first part of this review article will briefly survey some of the methodological issues and limitations related to noninvasive poststroke motor recovery studies. The second section will investigate some of the different mechanisms that underlie neurorehabilitation in recovering stroke patients. The third part will explore our current understanding of procedural memory processing and explore some of the neural regions that subserve motor memory consolidation and recent studies on reconsolidation in human subjects. Lastly, this article will explore some of the potential applications of integrating noninvasive tools with memory consolidation and reconsolidation theories to promote motor recovery. The overall objective of this work is to provide a better understanding of the compensatory mechanisms that are involved in poststroke motor recovery and neural regions engaged in motor memory formation in healthy adults, to demonstrate how noninvasive technologies have been utilized in the multidisciplinary field of clinical behavioral neuroscience, and to highlight their potential to be employed as clinical tools to promote individualized motor recovery in stroke patients.

## 2. PATIENT-RELATED LIMITATIONS ASSOCIATED WITH IMAGING TECHNIQUES

There are many important patient-related limitations to consider when using MRI. For example, in addition to general MRI contraindications (e. g., pacemaker, metallic implants, etc.), acute stroke (i.e., stroke onset within 24 hours) patients are more easily agitated, are medically unstable in consequence to underlying haemodynamic issues, are more likely to have a diminished level of consciousness associated with vomiting (i.e., increased risk of aspiration), and usually have other coexisting medical problems [4, 5]. In consequence to these issues and in order to investigate the neural mechanisms that drive recovery over and above the spontaneous time-dependent process, most fMRI studies are performed on the chronic (i.e., at least three months after stroke onset) stroke patient population and thus generalization may be limited to this cohort.

Another important patient-related methodological issue to consider is that fMRI studies may be confounded because when patients with vascular lesions are compared with normal controls, the blood oxygenation-level-dependent (BOLD) signal may reflect the underlying diseased hemodynamics and not differences in cortical activation. For example, the BOLD signal captured in recovering stroke patients is typically delayed and lower in amplitude, which could reflect disruptions in neurovascular coupling due to diffuse vascular disease [6].

## 3. fMRI METHODOLOGICAL ISSUES

Despite such limitations related to the patient population, fMRI offers excellent spatial (i.e., few millimeters) and temporal (i.e., few seconds) resolution [7–9]. In addition, early poststroke motor recovery fMRI studies have been designed as cross-sectional experiments that compared neural changes in fully recovered stroke patients and normal controls at a single point in time [7–9]. Such experiments have been limited because they do not show changes in neural activity throughout the duration of the recovery process. Recently, longitudinal studies have been designed to investigate neural activity in patients recovering from stroke over an extended period of time [7–9]. Such studies have allowed investigators to correlate changes in neural activation with specific motor gains during the recovery process.

Most studies have used a block design, whereby data is acquired in correlation to a distinct cognitive state (i.e., during a behavioral task) and then compared to a control period [7]. However, recent experiments have employed event-related design, whereby data is acquired during the repetition of discrete stimuli or responses (i.e., finger tapping task). In comparison to block design, the event-related paradigm has a longer acquisition time, but has certain advantages such as providing the option of using either periodic or randomized stimuli. Both block- and event-related designs enable continuous data collection usually with a repeat time of 2–5 seconds [7]. Such parameters must be taken into consideration in order to optimize data acquisition and derive meaningful results.

## 4. TMS METHODOLOGICAL ISSUES

TMS uses a strong but transient magnetic field that induces an electric current in the underlying cortical tissue [10, 12, 13]. Repetitive TMS involves repeated stimuli at intervals of 1–50 Hz for periods that range from 1–30 minutes [10, 12, 13]. Regular stimulation at low frequencies (upto 1 Hz) can inhibit cortical activation, whereas higher frequencies can stimulate cortical activity. TMS has been used to map functional cortical regions and inhibit or stimulate neural activity [10, 12, 13]. TMS is an excellent noninvasive instrument because it is of low cost, multifunctional, and relatively safe to the patient [10, 12, 13].

## 5. NEURAL SUBSTRATES THAT UNDERLIE COMPENSATORY MECHANISMS

Through the implementation of noninvasive techniques such as fMRI, investigators have been able to explore the neural regions that subserve compensatory mechanisms in recovering stroke patients. One important observation is that the integrity of the corticospinal tract, the main pathway from the cortical motor regions through the spinal cord to the muscles, correlates with functional motor recovery [14]. For example, Heald et al. [15] conducted a longitudinal study to evaluate the neurophysiological measurements of central motor conduction time (CMCT) immediately after stroke onset. They showed that normal or delayed CMCT correlated with a higher probability of survival and motor recovery. In addition, they found that patients with the poorest functional recovery at twelve months and greatest probability of stroke-related death responded least to intial cortical stimulation. In corroboration, Fujii and Nakada [16] demonstrated that the integrity of the ipsilesional sensorimotor cortex and corticospinal tract play an important role in motor recovery. They conducted an fMRI study in which they demonstrated that the rate of motor recovery, but not the absolute level of motor function, correlated with patterns of activation that were observed after subcortical stroke. Patients that recovered quickly, defined as within one month poststroke, showed similar patterns of activation in comparison to controls. However, patients that recovered more slowly, defined as within the end of the third month poststroke, showed greater activation in contralesional sensorimotor and supplementary motor areas. These studies suggest that patients with sufficient ipsilesional sensorimotor cortex and corticospinal tract integrity regain motor function more rapidly in comparison to patients with signifcant lesions.

There is also evidence that secondary cortical motor networks may play an important role in motor recovery. It has been suggested that motor function may be facilitated by projections from secondary motor regions and neural fibers originating in the primary motor region (M1), premotor cortex (PM), and supplementary motor area (SMA), which constitute parallel-independent motor networks with separate projections to spinal cord motor neurons and cortical regions [8, 17]. This implies that parallel motor networks could compensate for one another in consequence to neural damage [8]. In addition, Johansen-Berg et al. [20] illustrated through constraint-induced movement therapy (CIMT) that improvement in hand function correlated with increased fMRI activity in the ipsilesional premotor and somatosensory cortex; and Miyai et al. [21] reported increased ipsilesional premotor cortical (PM) activity in correlation with therapy-induced improvement in gait function.

Although such work does not directly prove that secondary motor regions subserve motor recovery, the application of TMS has been used to address this hypothesis more directly. Fridman et al. [22] applied TMS to four chronic stroke patients with focal subcortical lesions and showed that inhibition of the ipsilesional dorsal PM resulted in delays in a simple reaction time task, whereas TMS targeted to the contralesional dorsal PM of patients or in normal controls did not. In addition, Johansen-Berg et al. [23] showed that transient interference of the dorsal PM with TMS was more disruptive in patients with greater injury. In corroboration of both studies, Ward et al. [24] demonstrated that impaired functional integrity of the corticospinal system is associated with recruitment of bilateral secondary motor networks. Together, such work suggests that both secondary motor regions may also represent a compensatory pathway in motor recovery.

It is well established that the ipsilesional primary motor cortex plays an important role in motor recovery. The evidence surrounding the role of contralesional motor cortex remains controversial; however Chen et al. [25] demonstrated that temporarily inhibiting the contralesional M1 results in errors in both complex and simple motor tasks, suggesting that this region may play a role in planning and organizing motor movements [20]. In contrast, Johansen-Berg et al. [20] showed that disruption of the contralesional M1 using TMS does not impair performance in simple motor task, hence raising questions whether it subserves recovery after stroke. They also found that patients with moderate or poor outcome activate unaffected contralesional cortical motor regions more than those who recovered better. Furthermore, Murase et al. [26] has proposed that contralesional M1 may impair recovering motor function in patients with small subcortical stroke through interhemispheric inhibitory projections on ipsilesional M1 during attempted voluntary movement of the affected hand. More work is still needed, however, to better understand the role of the contralesional M1 in motor recovery.

Longitudinal studies have been designed to investigate the dynamic changes in neural regions in correlation to motor recovery. Calautti et al. [27] found that patients had greater bilateral activation in the sensorimotor region during paretic hand movement early after stroke onset in comparison to normal controls, but this pattern normalized in association with regained motor function (*~* 8 months poststroke). In addition, Feydy et al. [28] demonstrated patterns of activation correlated with location of stroke-induced lesions. They showed that after initial recruitment of bilateral areas, activation gradually shifted towards the ipsilesional sensorimotor cortex. Ward et al. [29] showed that patterns of activation increased in the sensorimotor area in stroke patients during paretic hand movement early after stroke onset, but this trend decreased toward a normal pattern in correlation with motor recovery. In addition, Zemke et al. [30] used fMRI to study recovering subcortical stroke patients while they performed a hand-grip task over six months. They found an initial overactivation within the primary and secondary motor regions, and characterized a focusing of task-related brain activation towards a more “normal” lateralized pattern. Taken together, these studies suggest that normalization of activation in the sensorimotor network, following early increased activation, also correlates with better motor recovery after stroke.

Other quantitative indices have demonstrated changes in neural activity in correlation to motor recovery. For example, Cramer et al. [31] showed that recovered stroke patients had a significantly lower laterality index (LI) = (C−I)/(C+I), where C = contralesional and I = ipsilesional regions, in comparison to controls. The LI can range from +1, which is exclusively ipsilesional, to −1, which is exclusively contralesional. Other studies have also shown that normalization in the LI shifts towards the ipsilesional sensorimotor networks in correlation with motor recovery after stroke [6, 32]. In addition, Marshall et al. [33] demonstrated the laterality of activation in primary sensorimotor cortex during paretic hand movement shifted towards the contralesional hemisphere within the first week of stroke onset but returned back to the ispsilesional hemisphere in correlation to good motor recovery (*~* three to six months after stroke onset). Jang et al. [34] showed that motor recovery (5–15 months poststroke) is correlated with a shift in laterality of primary sensorimotor cortex activation during paretic hand movement from nearly bilateral to strongly ipsilesional. These studies suggest that normalization of sensorimotor cortex laterality is again linked to good recovery of motor function after stroke.

## 6. NEURAL SUBSTRATES OF MOTOR MEMORY FORMATION

Neural regions that are reengaged after injury afford an evolutionarily adaptive process to provide a pathway for continued motor output. Similar functional plasticity is also observed in the procedural memory system. For example, after learning a sequence of finger movements (i.e., finger-to-thumb opposition task), the motor trace is believed to be processed offline, characterized by at least two distinct stages; an initial (i.e., within a single training session) fast learning stage measured by significant improvements in task performance, followed by a slower stage where further gains transpires over several sessions [35–38]. Within 6–8 hours an initially labile motor trace becomes resistant to interference from various amnesic agents (i.e., learning another motor sequence task) and eventually persists despite periods without practice. The process by which newly learned motor information is transformed from a labile state into a robust memory trace is referred to as consolidation and is subserved by specific neural structures and networks [35–38].

Noninvasive imaging techniques have been used to investigate the neural regions that participate in motor memory processing and consolidation. For example, Karni et al. used fMRI to study the changes in BOLD signals underlying motor skill learning [38]. They showed that after weeks of practicing finger-to-thumb sequences within a brief period of time, there was a noticeable enhancement of activation of the M1 region, which persisted for several months, and further suggests that motor sequence learning is subserved by a slowly evolving long-term experience dependent reorganization of the primary motor cortex. In addition, Shadmehr and Holcomb used fMRI to study the underlying neuroanatomical correlates of short-term motor skill learning [39]. They showed that during the earlier stages of motor learning there is a shift from prefrontal cortical regions to the premotor cortex, posterior parietal, and cerebellar structures. Both studies highlight that motor memory processing is subserved by functional reorganization of neural regions.

It has also been proposed that procedural memories can undergo a higher level memory formation process, referred to as system consolidation, whereby over longer periods of time, which can range from days to years pending on memory system, newly learned information is transferred from one neural processing region to another location for long-term storage [40]. For example, Doyon and Ungerleider [41] proposed that during the fast-learning stage, defined as a noticeable improvement in performance within a single training session, the corticocerebellar (CC) and corticostriatal (CS) systems are engaged pending on the motor skill learning task. However, when a motor sequence or adaptation task is well learned, the neural representations are thought to be distributed in one of two circuits, whereby the CS pathway supports the new motor sequence trace and the CC subserves motor adaptation [41, 42]. This theory was recently revised to incorporate findings that suggest that cerebral functional plasticity exists within the striatum and cerebellum in the later stages of motor sequence learning and motor adaptation, respectively [35]. For example, a 3T fMRI study that tracked motor sequence learning within the basal ganglia circuitry and motor-related structures showed improvements in task performance correlated with a change in signal from the associative to sensori-motor regions within the putamen, which suggests that this switch or transfer of information is functionally important for a motor memory trace to persist with time [43]. Understanding the neural regions that are engaged in motor memory formation may provide further insight into ways to enhance motor recovery in stroke patients.

Although noninvasive imaging techniques have provided much insight into the neural regions that underlie memory consolidation, our overall assumption that this process is permanent has been challenged [44–46, 49]. Misanin et al. used electroconvulsive shock treatments (ECST) to show that memory could be disrupted when in transition from a stored to an active state [45]. The process by which consolidated memories become labile and require stabilization after reactivation is now referred to as reconsolidation [44]. More recently, Nader et al. showed that the reactivation of a consolidated fear memory requires de novo protein synthesis in order for such information to persist with time [46]. Debiec et al. further expanded on these findings by demonstrating that hippocampal-dependent memories undergo both reconsolidation within the hippocampus, referred to as cellular reconsolidation, and at a second level of processing termed systems reconsolidation [47].

Whereas most work characterizing the reconsolidation of reactivated, once well consolidated, memories have been performed mostly on animals, there are ample publications showing the reconsolidation effect in human subjects. For example, Rubin reported that all twenty-eight psychiatric patients that received ECST after recall or recurrence of their psychiatric symptoms dramatically improved, some being symptom free when interviewed ten years after treatment [48]. In addition, Walker et al. utilized a motor skill finger tapping paradigm to show that overnight improvements in accuracy were significantly lost when a second interference trace (i.e., from a competing sequence task) was learned immediately after reactivating the first motor trace [49]. They showed that reactivating that trace one day after learning brought the trace back into a labile state that became sensitive to disruption from the interference trace. Although they failed to show the effect on their main dependent variable (i.e., the speed at which sequences are executed in a 30-second period), this study provides the first evidence that the reactivation of a stable and well-consolidated motor memory brings certain components back again into a labile state. More recently, Hupbach et al. showed in a group of college students that providing subjects with a reminder enhanced recall twenty-four hours after the reactivation of a consolidated list of items [50]. The latter finding suggests that reconsolidation may have a constructive effect on episodic memory processing. Although more work is necessary to further characterize and identify the boundary conditions associated with reconsolidation, it may be feasible to apply reconsolidation theory to enhance neurorehabilitation in stroke patients undergoing physical therapy.

## 7. FUTURE IMPLICATIONS FOR NOVEL THERAPY

How can our understanding of the neural substrates that subserve post-stroke compensatory mechanisms and procedural memory formation in healthy adults assist in clinical therapy? To a certain extent, most neurorehabilitation therapies consist of training patients how to perform previously learned tasks in a different way [3]. For example, arm ability training was developed for patients with mild hemiparesis and maximizes the retention and generalization of what is learned during the rehabilitation session through varying the difficulty of repetitive motor tasks [51, 52]. In addition, another behavioural therapy mentioned previously is CIMT, which has two components that are administered over two weeks. For example, the patient overcomes learned nonuse of the less functional extremity by practicing motor tasks for six hours per day while simultaneously restraining the use of the more functional extremity for 90% of the patient’s waking hours [52].

In both of these training protocols, success relies on learning novel ways to regain lost motor functions, which requires learning new procedural movements that are mediated by similar neural regions that drive procedural memory formation. Therefore, it is plausible that manipulation of these regions may enhance neurorehabilitation. For example, it has been proposed that the application of TMS to the nonaffected hemisphere could be employed to disrupt the interhemispheric inhibition that has previously been described [52–56]. In addition, TMS could also be used to target the affected hemisphere in order to stimulate regions that are damaged or enhance the neural substrates that underlie motor memory formation [52]. Although speculative, it is conceivable that inhibiting the reconsolidation of a nonfunctional motor memory trace via TMS may help a patient learn how to use a corresponding unaffected limb. Moreover, this may be facilitated through simultaneously enhancing the underlying neural substrates that promote forming functional motor memory traces via TMS. These procedures could be used in concert with other neurorehabilitation therapies in order to reprogram novel motor movements.

Numerous variables such as the neuroanatomical regions affected, the period of time since injury, and the patients’ previous experiences will all influence the neural substrates that are engaged after stroke [3, 8]. Thus, another potential application of fMRI could be used as a clinical tool to identify a patient’s specific *type* of neurocompensatory mechanism. This could enable physicians and rehabilitation specialists to tailor their treatment strategy to more accurately address their patient’s individual needs and requirements.

## 8. CONCLUSION

This review surveyed some of the literature which has examined the application of fMRI and TMS to study the neural substrates that underlie compensatory mechanisms in both stroke recovery and the neural regions that drive procedural memory formation in healthy adults. Although more work is necessary to further understand the mechanisms that subserve neural plasticity, the current literature suggests that specific neuroanatomical regions can be identified with fMRI and be stimulated or inhibited with TMS to cause functional changes in motor output. Such noninvasive tools may one day be more routinely applied to promote neurorehabilitation to benefit patients recovering from poststroke motor impairments.
